# Identification of a Metastasis-Associated Gene Signature of Clear Cell Renal Cell Carcinoma

**DOI:** 10.3389/fgene.2020.603455

**Published:** 2021-02-04

**Authors:** Suhua Gao, Lei Yan, Hongtao Zhang, Xiaoguang Fan, Xiaojing Jiao, Fengmin Shao

**Affiliations:** He'nan Provincial Key Laboratory of Kidney Disease and Immunology, Department of Nephrology, He'nan Provincial People's Hospital, People's Hospital of Zhengzhou University, Zhengzhou, China

**Keywords:** clear cell renal cell carcinoma, metastasis, biomarker, immune infiltration, tumor mutation burden

## Abstract

Clear cell renal cell carcinoma (ccRCC) is one of the most frequent pathological subtypes of kidney cancer, accounting for ~70–75%, and the major cause of mortality is metastatic disease. The difference in gene expression profiles between primary ccRCC tumors and metastatic tumors has not been determined. Thus, we report integrated genomic and transcriptomic analysis for identifying differentially expressed genes (DEGs) between primary and metastatic ccRCC tumors to understand the molecular mechanisms underlying the development of metastases. The microarray datasets GSE105261 and GSE85258 were obtained from the Gene Expression Omnibus (GEO) database, and the R package limma was used for DEG analyses. In summary, the results described herein provide important molecular evidence that metastatic ccRCC tumors are different from primary tumors. Enrichment analysis indicated that the DEGs were mainly enriched in ECM–receptor interaction, platelet activation, protein digestion, absorption, focal adhesion, and the PI3K–Akt signaling pathway. Moreover, we found that DEGs associated with a higher level of tumor immune infiltrates and tumor mutation burden were more susceptible to poor prognosis of ccRCC. Specifically, our study indicates that seven core genes, namely the collagen family (COL1A2, COL1A1, COL6A3, and COL5A1), DCN, FBLN1, and POSTN, were significantly upregulated in metastatic tumors compared with those in primary tumors and, thus, potentially offer insight into novel therapeutic and early diagnostic biomarkers of ccRCC.

## Introduction

Clear cell renal cell carcinoma (ccRCC) is one of the most aggressive histologic subtypes of kidney cancer, accounting for ~3% of all human cancers (Muglia and Prando, 2015). Up to 30% of ccRCC patients have metastases at the time of diagnosis, and ~60% have metastases within the initial 2–3 years after diagnosis (Casuscelli et al., 2017). Metastasis is the major reason for mortality associated with ccRCC. Although surgery is highly effective for the treatment of ccRCC (Chen et al., 2009), the treatment options available for patients with metastatic disease are very limited (Fisher et al., 2000; Flanigan et al., 2001).

Transcriptional profiling has emerged as an effective strategy to discover the molecular mechanisms underlying the metastasis or progression of ccRCC and predict clinical outcomes. While there have been comprehensive overviews of somatic mutations and transcriptomic profiles of primary ccRCC within The Cancer Genome Atlas project (The Cancer Genome Atlas Research Network, 2013), the genomic and transcriptomic profiles of metastatic ccRCC have not been examined in the context of their primary tumors.

Immunotherapy has recently been identified as an effective methodology for advanced or aggressive cancers (Hoos, 2016; Aoun et al., 2017; Kamal et al., 2018). In addition, many studies have found that the tumor mutation burden (TMB) and neoepitopes in many cancer types are closely associated with immunotherapy (Kandoth et al., 2013; Brown et al., 2014). However, few relevant research studies have centered on the correlation of the TMB with immune infiltrates and tumor metastasis in ccRCC. Therefore, we identified a metastasis-associated gene signature that supports ccRCC metastases by comparing gene expression profiles, the TMB, and immune infiltrate differences between metastatic and primary tumors.

## Materials and Methods

### Data Collection and Identification of DEGs

We searched the GEO (Gene Expression Omnibus) database (https://www.ncbi.nlm.nih.gov/geo/) using the following keywords: “Clear cell renal cell carcinoma” AND “Primary” AND “Metastatic” AND “*Homo sapiens*” AND “Expression profiling by array.” After a systematic review, two gene expression profiles (GSE105261 and GSE85258) were collected for analysis. Then, the R package limma was used for DEG analysis. We perceived *p* < 0.05 and a |log (FC, fold change)| >1 to be statistically significant for the DEGs, and logFC ≥1 and logFC ≤-1 were used to indicate upregulated and downregulated DEGs, respectively. Using all of the DEGs identified in GSE105261 and GSE85258, we constructed a volcano plot with the R package ggplot2. The resulting dataset of DEGs was gathered and used for further analyses.

### PPI Network Construction and Analysis of Clusters

The STRING database (http://string-db.org/) is an online database designed to provide a vital assessment and integration of protein–protein interactions, which includes direct (physical) and indirect (functional) associations (von Mering et al., 2003). Cytoscape and Gephi are popular open-source software devices for the visual exploration of biomolecule interaction networks composed of proteins and genes and other types of interactions (Bastian et al., 2009; Smoot et al., 2011). The DEGs were mapped with STRING to evaluate the protein–protein interactions (PPIs) and then visualized with Cytoscape and Gephi. Then, the Molecular Complex Detection (MCODE) plugin was used to screen core cluster from the PPI network with degree cutoff = 2, node score cutoff = 0.2, haircut = true, fluff = false, K-Core = 2, and max. depth from seed = 100.

### Identification and Analysis of Shared DEGs and Hub Genes

By writing an R script, we compared the DEGs of two GSE samples and identified the shared DEGs (including upregulated, downregulated, and reversed expressions). Then, we employed unsupervised hierarchical clustering and expression correlation calculations based on the shared DEG series matrix file and plotted them with the R package ggplot2. The Annotation, Visualization, and Integrated Discovery Database (DAVID, v6.8, https:/david.ncifcrf.gov/) was employed to conduct Gene Ontology and Kyoto Encyclopedia of Genes and Genomes pathway enrichment analyses. A modified Fisher's exact test, the *p*-value (or EASE score), was used to examine the significance of gene ontology/KEGG pathway term enrichment. The Benjamini–Hochberg procedure was used to correct the *p*-values of individual term member enrichment globally. These gene ontology/pathway terms with a *p*-value cutoff ≤0.05 and Benjamini–Hochberg cutoff ≤0.5 were regarded as significant and interesting.

In the present study, the PPI network of shared DEGs was constructed using the STRING database, and interaction with a combined score >0.4 was regarded as statistically significant. Subsequently, engaging the Network Analyzer plugin in Cytoscape, the network topology parameters were analyzed to obtain the average shortest path length (ASPL), betweenness centrality (BC), etc., and nodes with a shorter ASPL and higher BC were considered as hub genes (Assenov et al., 2008; Li et al., 2018).

### Validation of Shared DEGs

To validate the mRNA expression level of the identified shared genes, the ONCOMINE microarray database (https://www.oncomine.org), which is a translational bioinformatics service that provides a powerful genome-wide expression analysis (Rhodes et al., 2004) was used. Data were extracted to assess the mRNA expression levels (cancer vs. normal) of shared DEGs in multiple types of cancer, including ccRCC. In this study, the thresholds were set as *p* < 0.05, a fold change of 2, and a gene ranked in the top 10%. Student's *t*-tests were used to analyze the expression differences.

### Determination of Shared DEG Alterations in ccRCC

The data were obtained from cBioPortal (http://www.cbioportal.org/), an open-access platform for assessing genetic mutation variability among pan-cancer patients (Gao et al., 2013). cBioPortal evaluated the frequency of genetic alterations (including mutations, amplifications, deletions, and associations of fusions with clinical parameters) across ccRCC studies.

### Survival Analysis of Shared DEGs

We used the Kaplan–Meier (KM) plotter (http:/kmplot.com/analysis/) to perform analysis of the shared DEGs with KIRC (kidney-clear cell carcinoma) overall survival (OS). In this study, patients were divided by autoselection best cutoff, type of cancer = kidney clear cell carcinoma (*n* = 530), and survival = OS (*n* = 7,642) as the basic parameters. Based on the gene transcriptional expression level of a given gene, the plotter endows users with the ability to separate patients into high and low expression groups and create KM plots. In addition, the hazard ratio (HR) was calculated and shown on the chart with 95% confidence interval and the log-rank *p*-value, and the number-at-risk is shown below the curves.

### Tumor-Infiltrating Immune Cell Association With Gene Expression in Tumor Immune Estimation Resource

The association between the abundance of immune tumor infiltrates (B cells, CD4^+^ T cells, CD8^+^ T cells, dendritic cells, macrophages, and neutrophils) and the expression of the selected genes was analyzed *via* the Tumor Immune Estimation Resource (TIMER) platform, a web server that contains 10,897 samples of various types of cancer available in the TCGA database (Li et al., 2017). The gene module allows users to select any gene of interest and visualize its expression in correlation with the level of immune infiltration in different types of cancer. The scatterplots of correlation showed the value of the partial Spearman correlation, corrected by purity, and its statistical significance.

## Results

### Identification of DEGs

**Figure 1:**
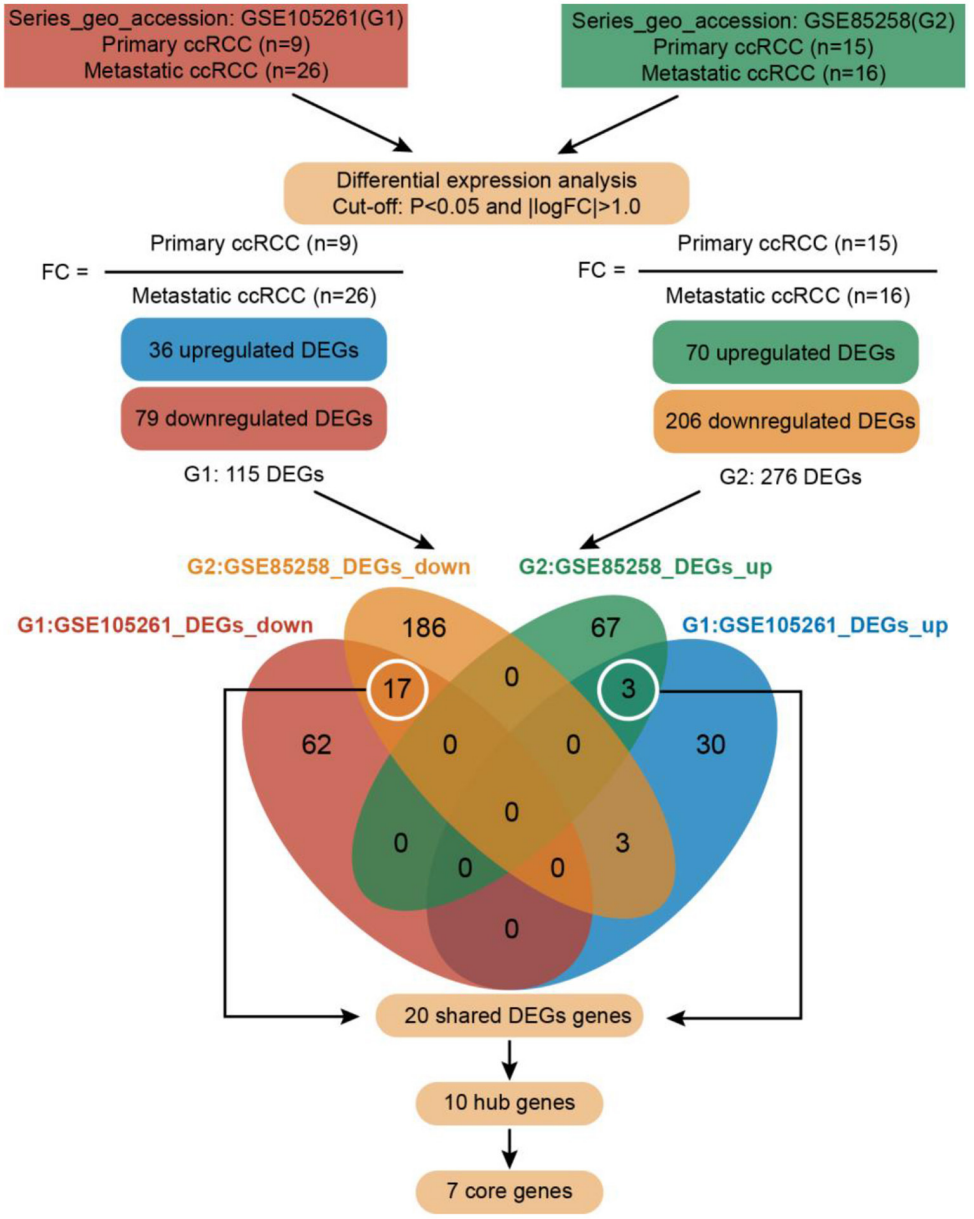
Roadmap of the approach and summarized findings.

**Figure 2:**
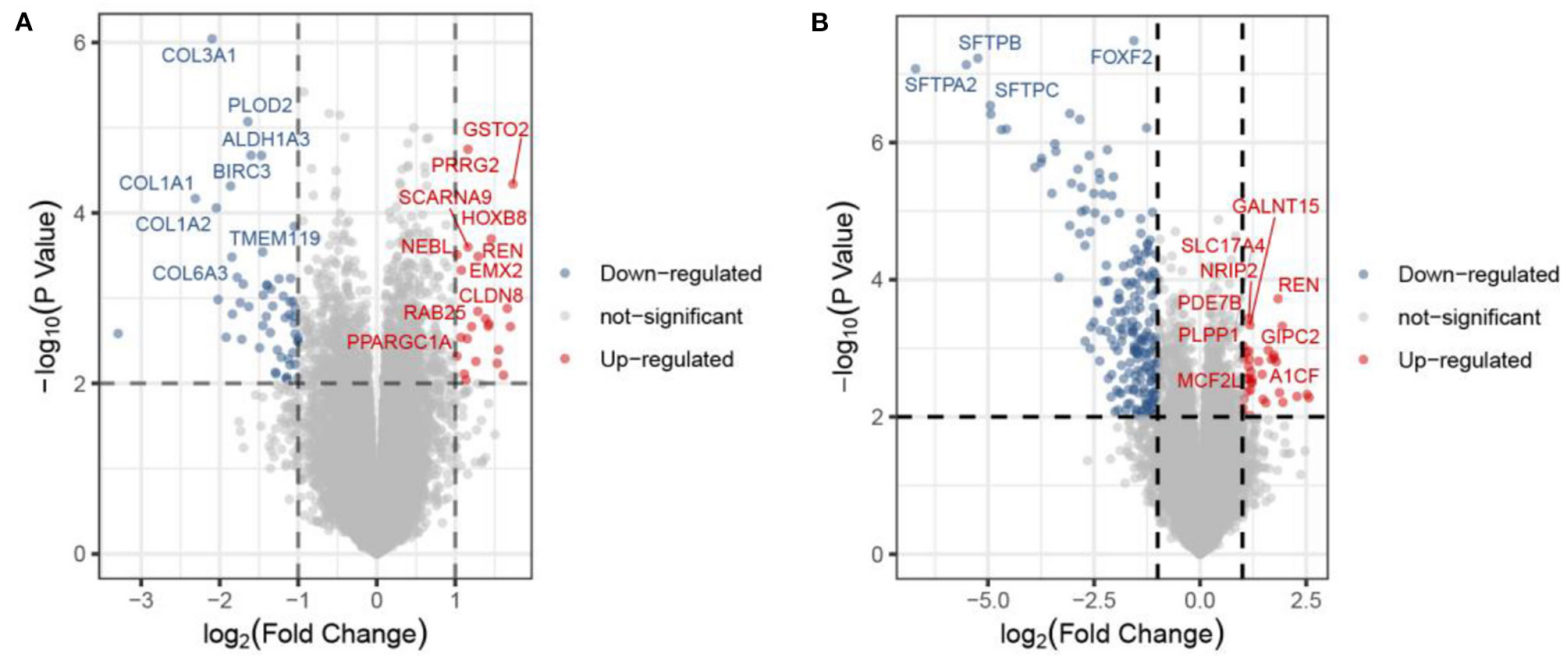
Volcano plot of the DEGs in the primary ccRCC group compared with the metastatic ccRCC group from the GSE105261 **(A)** and GSE85258 **(B)** datasets. Each point corresponds to one gene.

The methodology roadmap for our study is shown in Figure 1. In summary, we obtained the gene expression profiles for GSE105261 and GSE85258 from the GEO database. The sample information of GSE is shown in Table 1. Overall, 115 and 276 DEGs were identified from the GSE105261 and GSE85258 datasets, respectively. Furthermore, 20 shared DEGs (17 downregulated, three upregulated) of the two groups were identified through multiple comparisons, and 10 genes sorted by the integrated score were considered as hub genes (Tables 2, 3). The results are shown in Figure 1 and Supplementary Material 1. We constructed a volcano plot with the R package ggplot2 (Figure 2).

**Table 1:** Characteristics of the GSE105261 and GSE85258 datasets.

**Series code**	**Series geo accession**	**Series type**	**Number of samples**	**Group**	**Organism**	**Series platform id**
G1	GSE105261	Expression profiling by array	35	Primary ccRCC (*n* = 9) Metastatic ccRCC (*n* = 26)	*Homo sapiens*	GPL10558
G2	GSE85258	Expression profiling by array	31	Primary ccRCC (*n* = 15) Metastatic ccRCC (*n* = 16)	*Homo sapiens*	GPL570

**Table 2:** The shared DEGs of the GSE105261 and GSE85258 datasets.

	**Gene**	**Regulation**	**Gene title**
1	REN	Up	Renin
2	OGDHL	Up	Oxoglutarate dehydrogenase-like
3	HSD11B2	Up	Hydroxysteroid 11-beta dehydrogenase 2
4	COL3A1	Down	Collagen type III alpha 1 chain
5	COL1A1	Down	Collagen type I alpha 1 chain
6	COL1A2	Down	Collagen type I alpha 2 chain
7	COL6A3	Down	Collagen type VI alpha 3 chain
8	PRRX1	Down	Paired related homeobox 1
9	POSTN	Down	Periostin
10	COL5A1	Down	Collagen type V alpha 1 chain
11	FBLN1	Down	Fibulin 1
12	SPINK13	Down	Serine peptidase inhibitor, Kazal type 13 (putative)
13	GJB2	Down	Gap junction protein beta 2
14	PDGFRL	Down	Platelet-derived growth factor receptor-like
15	CTHRC1	Down	Collagen triple helix repeat containing 1
16	FGG	Down	Fibrinogen gamma chain
17	SCG5	Down	Secretogranin V
18	DCN	Down	Decorin
19	LUM	Down	Lumican
20	MXRA5	Down	Matrix remodeling associated 5

**Table 3:** The top 10 hub genes in the shared DEG PPI network.

**Rank**	**Average shortest path length**	**Betweenness centrality**	**Degree**	**Symbol**
1	1.08333333	0.05176768	11	COL1A2
2	1.08333333	0.05176768	11	COL1A1
3	1.08333333	0.05176768	11	COL3A1
4	1.08333333	0.05176768	11	POSTN
5	1.25	0.01515152	9	LUM
6	1.25	0.01515152	9	COL6A3
7	1.25	0.01010101	9	COL5A1
8	1.25	0.16666667	9	DCN
9	1.25	0.01010101	9	FBLN1
10	1.58333333	0	6	MXRA5

### Construction of the PPI Network and Clusters

**Figure 3:**
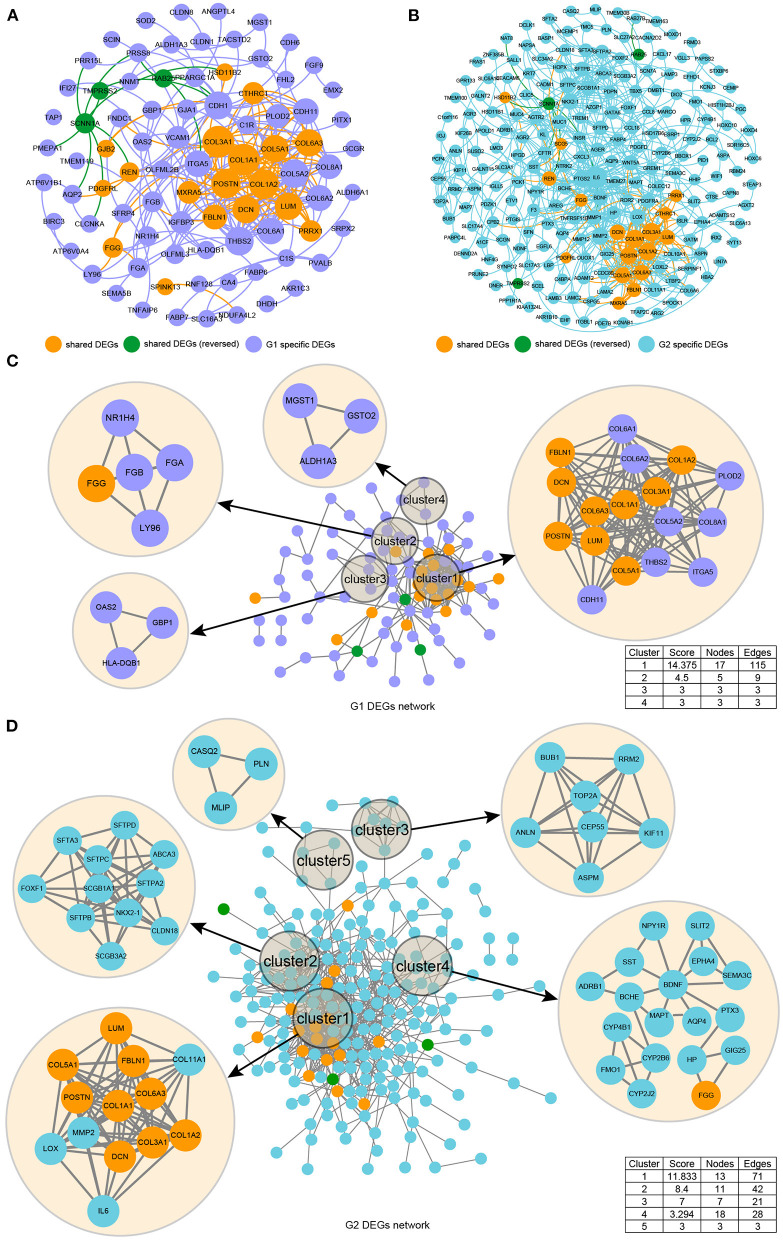
Illustration of the protein networks of DEGs from the GSE105261 (G1) and GSE85258 (G2) datasets. The spatial distribution characteristics of the PPI network model of G1 **(A)** and G2 **(B)** based on a macro perspective were constructed by the “Fruchterman–Reingold” layout in the Gephi software. The spatial distribution characteristics of the PPI network model of G1 **(C)** and G2 **(D)** based on independent perspectives were constructed by the MCODE plugin of Cytoscape software.

We used STRING to identify the PPI networks for both the up- and downregulated genes to assess the PPIs between the DEGs. A combined score of ≥0.9 was considered to indicate a significant interaction. Then, we focused on exploring the spatial distribution characteristics of 20 shared DEGs in the PPI network model of the two datasets to verify the reliability. We exported the resulting PPI network from STRING as a “CSV” file and imported it to visualization software Cytoscape v3.7.1 and Gephi 0.9.2. The graphical representations of the PPI networks are shown in Figure 3. The G1 and G2 DEG PPI network models comprised 87 nodes and 262 edges and 220 nodes and 634 edges, respectively. The results are presented in Supplementary Material 2. We found that the 20 shared DEGs were located in different spatial positions in the PPI network of the two sets of DEGs, but they were vital ones (Figures 3A,B). Then, we carried out independent cluster analysis on the two PPI networks, and we found that 20 shared DEGs were all distributed in the core cluster, suggesting that these genes were stable and reliable (Figures 3C,D).

### Validation of the mRNA Expression of Shared DEGs

**Figure 4:**
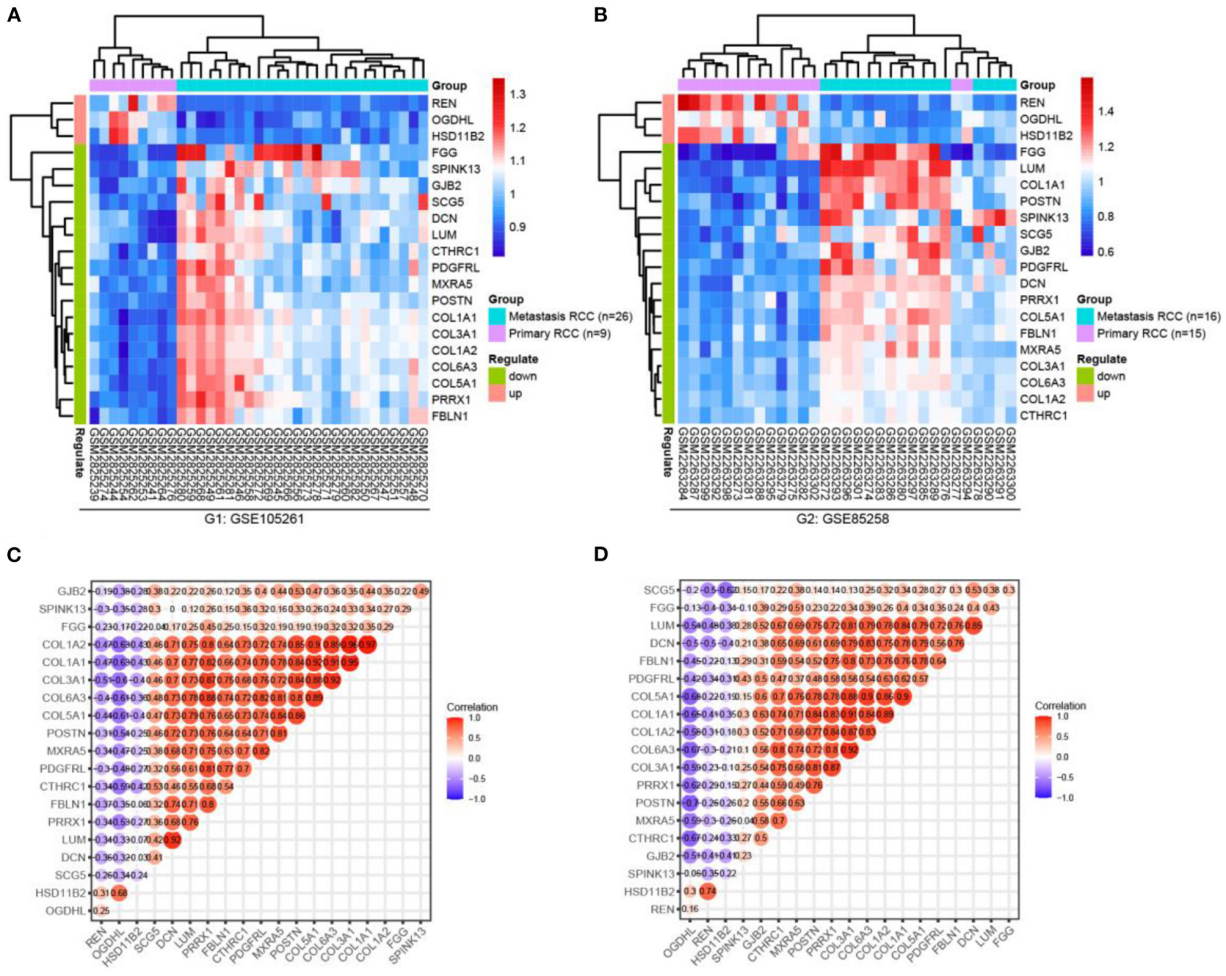
Hierarchical clustering analysis and expression correlation calculation of mRNA expression of shared DEGs, which were compared primarily with the metastatic ccRCC group in the GSE105261 **(A,C)** and GSE85258 **(B,D)** datasets.

**Figure 5:**
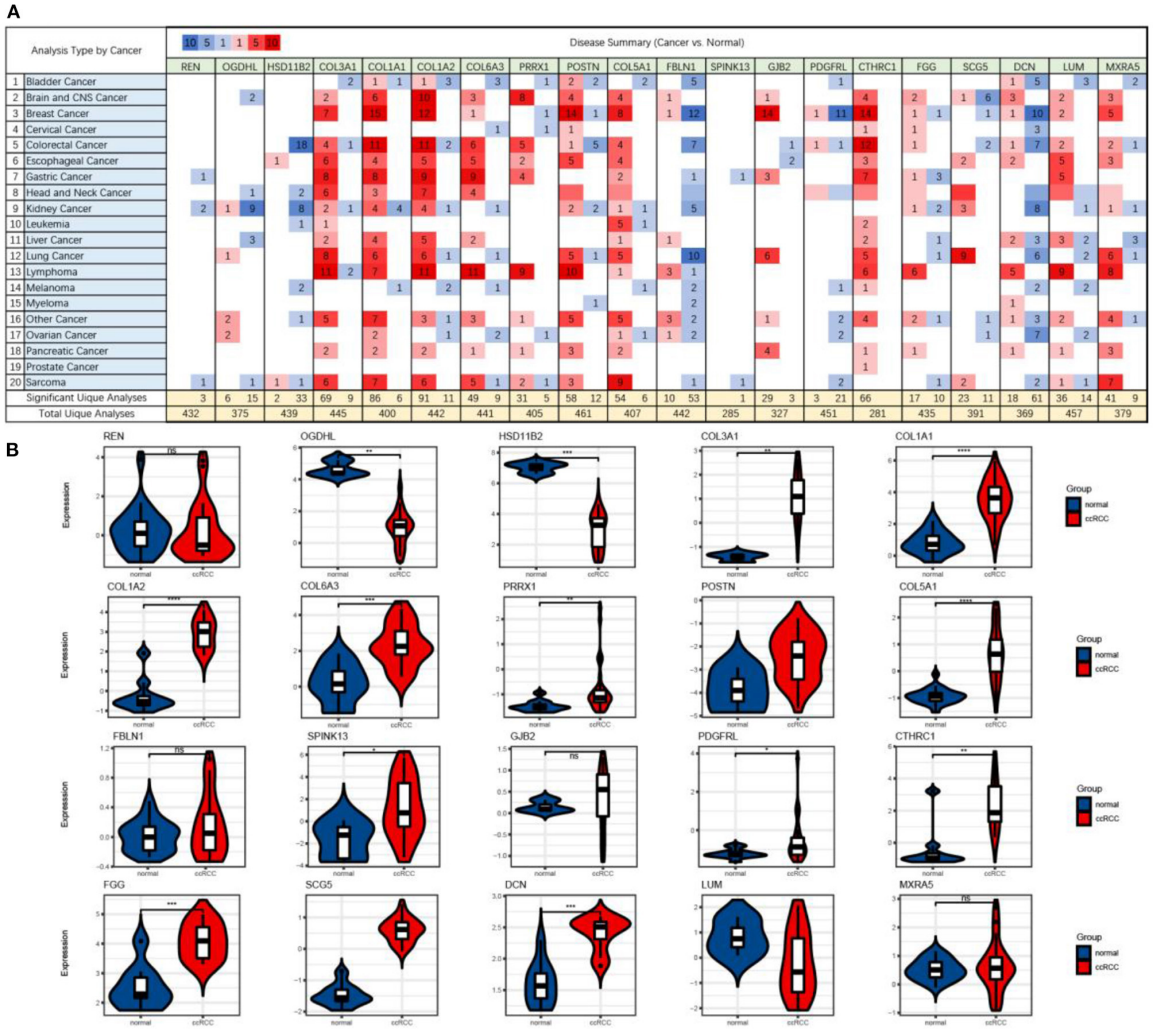
The mRNA expression levels (cancer vs. normal) of shared DEGs in multiple cancer types **(A)** and ccRCC **(B)**, which were based on ONCOMINE. The figure shows the numbers of datasets with statistically significant upregulated (red) and downregulated (blue) mRNA expression.

First, we checked the mRNA expressions of the GSE105261 and GSE85258 datasets and employed unsupervised hierarchical clustering and expression correlation calculations based on the shared DEG series matrix file. As demonstrated in Figure 4, the mRNA expression was obviously clustered into different groups (primary and metastatic) and different expression differences (downregulated and upregulated). Then, we further verified the mRNA expression levels of shared DEGs between multiple cancer types (Figure 5A) or ccRCC (Figure 5B) with non-tumor kidney tissues (normal group) using the ONCOMINE database. The mRNA expression levels of COL1A1, COL1A2, COL6A3, PRRX1, POSTN, COL5A1, SPINK13, PDGFRL, CTHRC1, FGG, SCG5, and DCN were markedly upregulated in ccRCC tissues (*p* < 0.05) compared with those in non-tumor kidney tissues.

### GO and KEGG Enrichment Analyses

**Figure 6:**
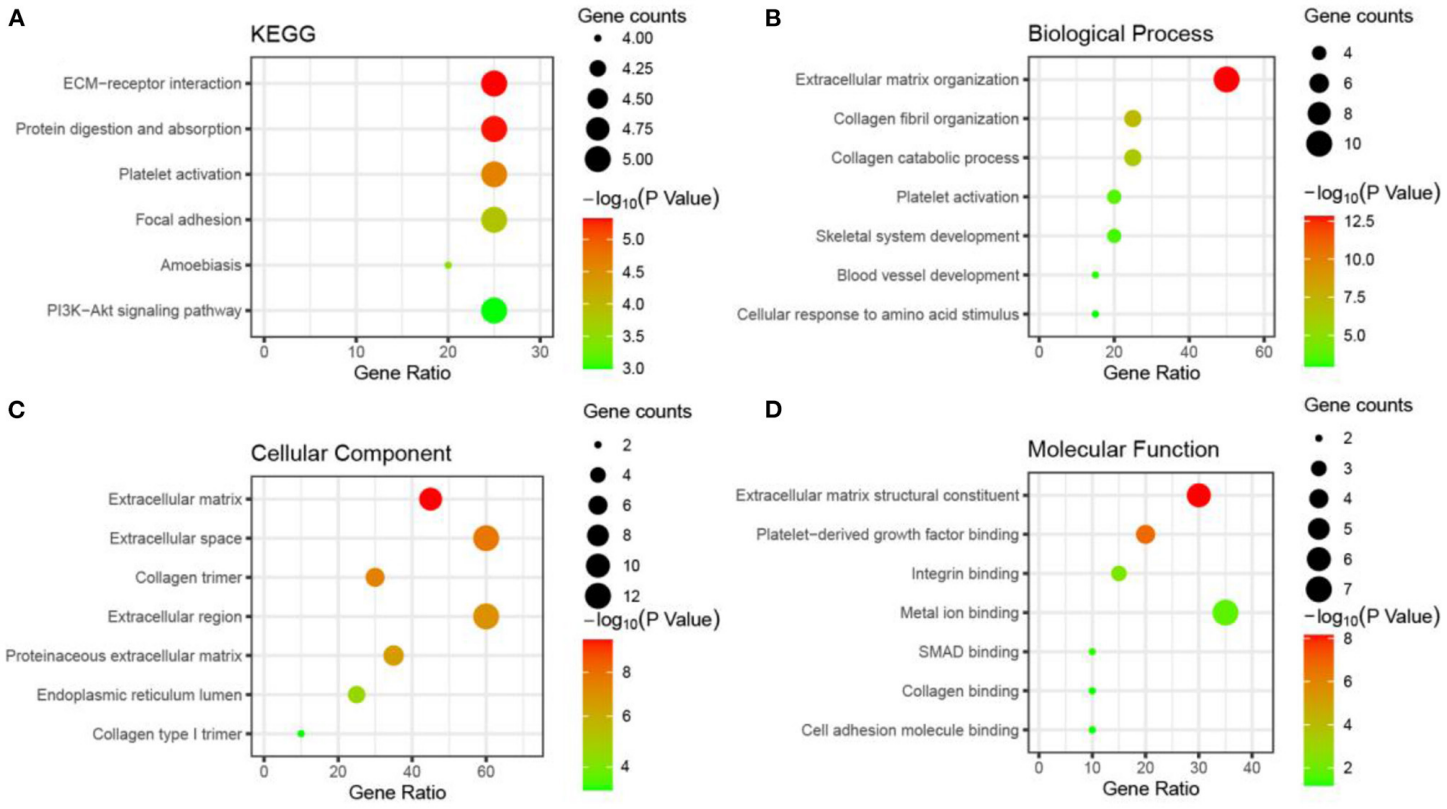
Gene ontology and KEGG pathway enrichment bubble diagram for shared DEGs (showing the first seven items).

We imported all shared DEGs into the online analytics tool DAVID to conduct the annotation process to determine the potential GO classifications and KEGG pathway-enriched genes from the dataset. The results are presented in Supplementary Material 3. The annotated results for the GO terms were divided according to the MF (molecular function), BP (biological process), and CC (cell component) categories (*p* < 0.05, FDR < 0.05). The results of the GO biological process analysis revealed that the shared DEGs were mainly enriched in the organization of extracellular matrix and collagen fibrils, collagen catabolic process, platelet activation, and the development of blood vessels (Figure 6). For the GO molecular function analysis, the shared DEGs were significantly enriched in platelet-derived growth factor, collagen, and cell adhesion molecule. The shared DEGs were mostly enriched in the extracellular matrix and the extracellular region in GO cell component analysis. By examining the KEGG pathways, we noticed an enrichment of the shared DEGs in platelet activation, protein digestion, absorption, focal adhesion, and the PI3K–Akt signaling pathways. These results suggested that the shared DEGs could be significantly related to the process of tumor aggression and immune infiltration.

### Mutation Burden and Selection Analysis

**Figure 7:**
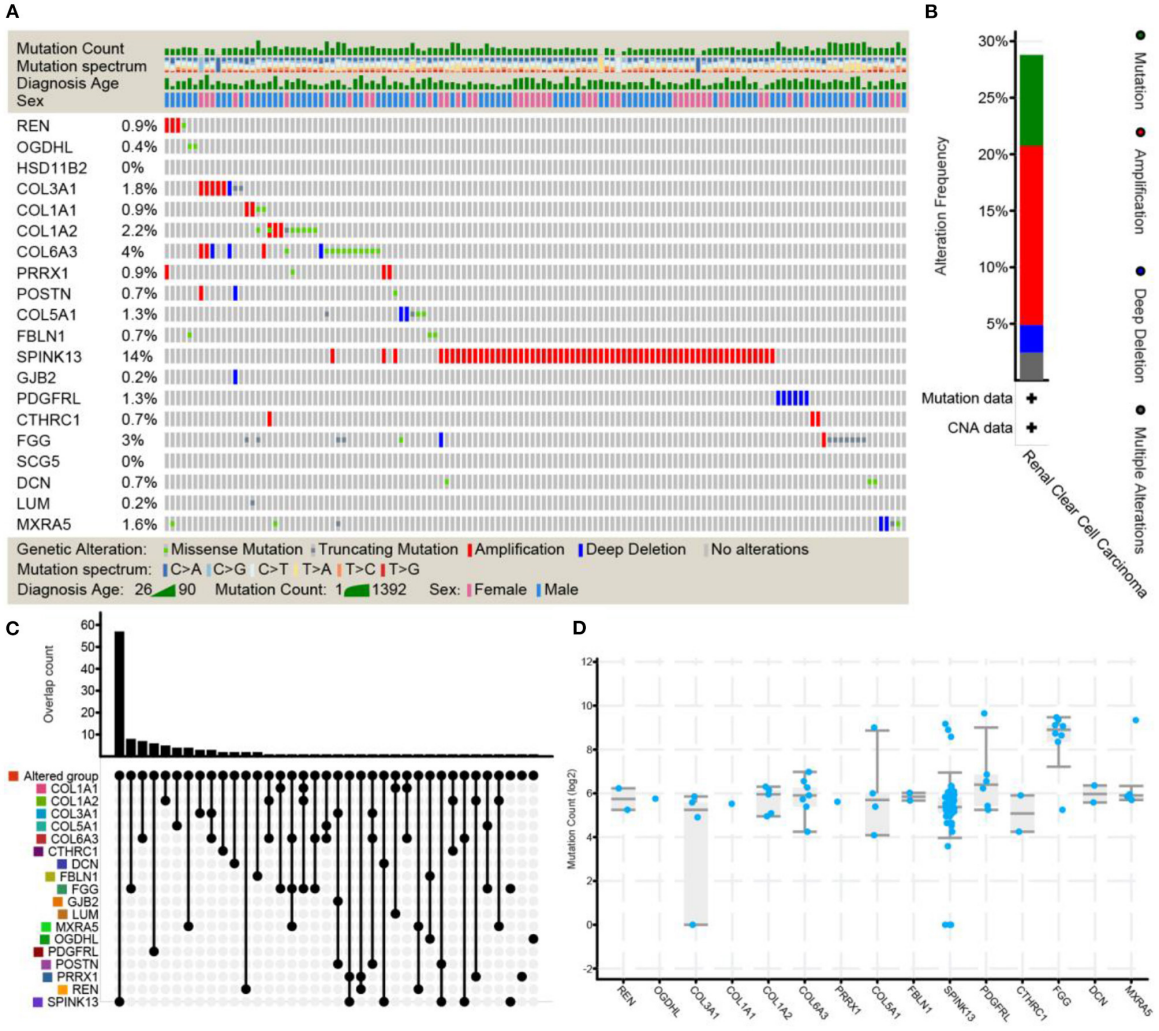
The frequency of genetic alterations (including mutations, amplifications, deletions, and fusions associated with clinical parameters) of shared DEGs was evaluated through ccRCC studies using cBioPortal. Stacked plots show mutational burden (histogram, top), mutations in shared DEGs (tile plot, middle), and mutational marks (bottom) **(A)**. Overall description and cancer type summary of the selected sample **(B)**. The histogram combined with the dotted-line graph shows the overlap of samples (patients) **(C)**. Box plots display shared DEG mutation counts based on the Kruskal–Wallis test (*p* = 0.0140) **(D)**. HSD11B2, POSTN, GJB2, SCG5, and LUM completely overlapped with other selected groups and were excluded from the analyses in other tabs.

The cBioPortal platform was used to identify mutational processes by adjusting the mutational signatures published on the platform to the mutational profiles of the somatic SNVs in ccRCC tumors, referring to the total number of genetic mutations per patient identified and patient survival. As shown in Figure 7, neither HSD11B2 mutations nor SCG5 mutations were identified. In addition, other shared DEGs were assessed for the existence of genetic alterations (including mutations, amplifications, deletions, and fusions associated with clinical parameters).

### Survival Analysis of Shared DEGs

**Figure 8:**
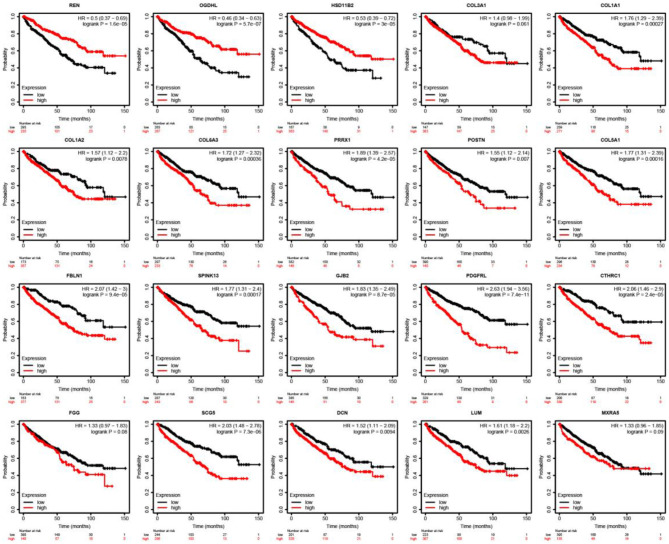
Outcomes associated with mutation and immune infiltrates were illustrated in patients with ccRCC. Kaplan-Meier survival curves with the log-rank test and hazard ratio (HR) for overall survival are shown.

The Kaplan–Meier test and Cox regression analysis were used to assess associations with OS (Figure 8). As a result, we noticed that a higher expression of REN (HR = 0.5; CI = 0.37–0.69; log-rank *p* = 1.6e-05), OGDHL (HR = 0.46; CI = 0.34–0.63; log-rank *p* = 5.7e-07), and HSD11B2 (HR = 0.53; CI = 0.39–0.72; log-rank *p* = 3e-05) was associated with the improved overall survival in ccRCC patients. The expression levels of REN, OGDHL, and HSD11B2 were higher in the primary group than those in the metastatic group.

However, the higher expressions of COL1A1 (HR = 1.76; CI = 1.29–2.39; log-rank *p* = 0.00027), COL1A2 (HR = 1.57; CI = 1.12–2.2; log-rank *p* = 0.0078), COL6A3 (HR = 1.72; CI = 1.72–2.32; log-rank *p* = 0.00036), PRRX1 (HR = 1.89; CI = 1.39–2.57; log-rank *p* = 4.2e-05), POSTN (HR = 1.55; CI = 1.12–2.14; log-rank *p* = 0.007), COL5A1 (HR = 1.77; CI = 1.31–2.39; log-rank *p* = 0.00016), FBLN1 (HR = 2.07; CI = 1.24–3; log-rank *p* = 9.4e-05), SPINK13 (HR = 1.77; CI = 1.31–2.4; log-rank *p* = 0.00017), GJB2 (HR = 1.83; CI = 1.35–2.49; log-rank *p* = 8.7e-05), PDGFRL (HR = 2.63; CI = 1.94–3.56; log-rank *p* = 7.4e-11), CTHRC1 (HR = 2.06; CI = 1.46–2.9; log-rank *p* = 2.4e-05), and SCG5 (HR = 2.03; CI = 1.48–2.78; log-rank *p* = 7.3e-06) were linked with worse overall survival in ccRCC patients. The expression of these genes, as listed above, was upregulated in the metastatic group compared with that in the primary group.

### Correlation of Hub Genes With Tumor Immune Infiltrates

**Figure 9:**
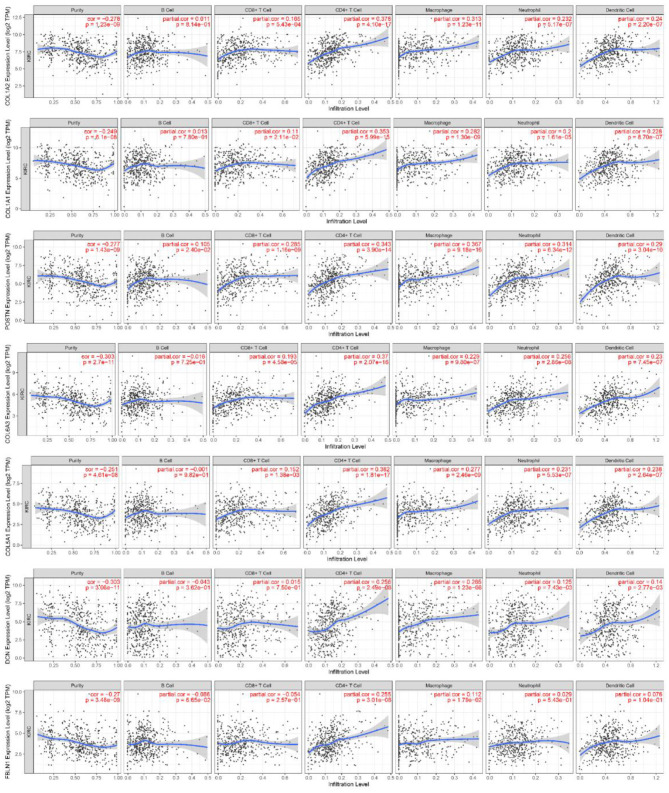
Association of core gene expression in ccRCC with immune infiltrates. Partial correlation analysis of gene expression and tumor immune infiltrate levels (B cells, CD8^+^ T cells, CD4^+^ T cells, macrophages, neutrophils, and dendritic cells).

Finally, we further explored the association between the expression of hub genes with worse overall survival and immune tumor infiltrates. The levels of the gene expression to the purity of the tumor are always displayed on the leftmost panel. As shown in Figure 9, tumor purity was negatively correlated with the expression levels of these genes (tumor purity, cor < 0). Genes highly expressed in the microenvironment were expected to have negative associations with tumor purity, while genes highly expressed in tumor cells were expected to have the opposite association (Alcaraz-Sanabria et al., 2020). Besides, the COL1A2 expression level correlated with the infiltration level of CD4^+^ T cells (part.cor = 0.378) and macrophages (part.cor = 0.313); the COL1A1 expression level correlated with the infiltration level of CD4^+^ T cells (part.cor = 0.353); the COL6A3 expression level correlated with the infiltration level of CD4^+^ T cells (part.cor = 0.37); the COL5A1 expression level correlated with the infiltration level of CD4^+^ T cells (part.cor = 0.382); the DCN expression level correlated with the infiltration level of CD4^+^ T cells (part.cor = 0.256) and macrophages (part.cor = 0.265); the FBLN1 expression level correlated with the infiltration level of CD4^+^ T cells (part.cor = 0.255); and finally, the POSTN expression level correlated with the infiltration level of CD4^+^ T cells (part.cor = 0.343) and macrophages (part.cor = 0.367). Therefore, these seven hub genes (COL1A2, COL1A1, COL6A3, COL5A1, DCN, FBLN1, and POSTN) were considered as core genes.

## Discussion

Though metastatic ccRCC treatment options have increased over the past decade, mortality and 5-year survival remain unsatisfactory (Courtney and Choueiri, 2010; Pal et al., 2012). Previous studies focused mainly on the screening of biomarkers expressed differently between tumor and normal tissues. In the more lethal and therapeutically relevant distant metastatic tumor, however, less is known regarding gene expression profiles. Microarray technology is one of the leading approaches that many researchers worldwide use to explore the gene expression levels involved in cancer (Russo et al., 2003; Perez-Diez et al., 2007). Hence, it is relatively more meaningful to survey the expression profiles of DEGs and predict metastasis-associated gene signatures. In this study, data were obtained from the GEO database from a total of 24 patients with primary ccRCC and 42 patients with metastatic ccRCC. For screening DEGs, we considered *p* < 0.05 and log (FC, fold change) > 1 to be statistically significant. As a result, a total of 115 and 276 DEGs, including 106 upregulated and 285 downregulated genes, were identified from the GSE105261 and GSE85258 datasets, respectively.

Good efficiency has been demonstrated in *in silico* methods, and network analysis has been shown to be a reliable way of depicting genomic data (Jeong et al., 2015). For large PPI networks, the topological interpretation of shared DEGs was required and was thus substantially based on integrated local components, such as the degree distribution node, the topological coefficient, the average shortest path length, the centrality of betweenness, and the centrality of closeness (Assenov et al., 2008). These parameters were used to analyze the nodes in individual PPI networks of the DEG dataset to determine their significance in networks with different characteristics. Then, we compared the DEGs of two GSE samples and identified 20 shared DEGs (namely three upregulated and 17 downregulated genes), and 10 hub genes were screened by constructing a PPI network. We found that the 20 shared DEGs were located in different spatial positions in the PPI network of the two sets of DEGs, but they were all vital ones (Figures 3A,B). Moreover, we carried out independent cluster analysis on the two PPI networks, and we found that 20 shared DEGs were all distributed in the core cluster, suggesting that these genes were stable and reliable (Figures 3C,D).

The focus of our further analyses was directed toward the validation of the global gene expression of shared DEGs. We first analyzed the transcriptional profiles of the GSE105261 and GSE85258 datasets. Hierarchical clustering showed a perfect distinction between the primary and metastatic groups (Figure 4). Then, we further identified the mRNA expression levels of shared DEGs between multiple cancer types (Figure 5A) or ccRCC (Figure 5B) and non-tumor kidney tissues (normal group) based on ONCOMINE. Based on these results, it was revealed that the mRNA expression levels of shared DEGs distinguished metastatic ccRCC tissues from primary ccRCC tissues. These findings were consistent with the obtained microarray data.

Furthermore, we employed DAVID to implement GO and KEGG pathway enrichment analyses to determine MF, BP, and CC terms and pathways involving shared DEGs. The results indicated an enrichment of the shared DEGs in extracellular matrix (ECM)–receptor interaction, platelet activation, protein digestion and absorption, focal adhesion, and the PI3K–Akt signaling pathway (Figure 6). These pathways were reported to promote the migration and invasion of cancer cells (Northey et al., 2017; Stein et al., 2019). The ECM consists of a complex mixture of structural and functional macromolecules and plays an important role in tissue and organ morphogenesis and in maintaining the structure and function of cells and tissues. The pathway of ECM–receptor interactions leads to direct or indirect control of cellular activity, such as adhesion, migration, differentiation, proliferation, and apoptosis. Cancer cell activation of platelets has a myriad of procancer effects, such as stimulating tumor growth, preparing the metastatic niche, and helping metastatic cells survive in circulation (Gay and Felding-Habermann, 2011). The phosphoinositide 3-kinase (PI3K)/Akt pathway is a classic and important signaling pathway that is involved in numerous cellular functions, including cell proliferation, survival, adhesion, migration, and metabolism (Xu et al., 2016; Yin et al., 2017). Therefore, our observed results were consistent with the role of shared DEGs in tumor aggressiveness pathways and abnormal cell cycle and mitosis functions. Based on the above results, these shared DEGs partially represent metastasis-specific genes and showed significant biological progression of the tumor and may contribute to the progression toward increased malignancy.

It is widely known that the tumor mutation burden is an important indicator of immunotherapy (Foulkes et al., 2016; Samstein et al., 2019). In this study, we showed that there was a significantly higher mutational burden associated with worse overall survival. Although prior work has shown an association between the number of mutations and outcomes in ccRCC, no distinctions have been made between patients who were diagnosed with metastatic disease and patients who were diagnosed with localized disease (Hsieh et al., 2015, 2017). However, those tumors that progress at relapse or metastatic sites may have accumulated additional genomic mutations over time, and this hypothesis is supported by data from other cancer subtypes (Yates et al., 2017).

Given that patients with metastatic ccRCC have poor prognosis, we decided to investigate the capacity of metastasis-associated gene signatures to predict the overall survival in patients with ccRCC tumors. As a result, we reported a clear association of our gene signatures with a favorable prognosis that was higher than those of metastatic cancer. Specifically, the higher expression of COL1A2, COL1A1, COL6A3, COL5A1, DCN, FBLN1, and POSTN in patients with ccRCC was associated with immune infiltrates and worse overall survival (Figures 8, 9). Moreover, the existence of genetic alterations (including mutations, amplifications, and deletions) of these genes was assessed (Figure 7). Solid tumors have been reported to consist of cancer cells that interact with the tumor microenvironment, which includes stromal cells, immune cells, and ECM, and poor prognosis of breast, gastric, and oral cancers (Ohno et al., 2010; Conklin et al., 2011; Li et al., 2013). Several studies suggest that POSTN (periostin) can act to promote cell migration by facilitating the interaction between cancer cells and the tumor niche. These interactions are essentially mediated through interactions with integrin family receptors (Laura and Javier, 2018). The results showed that immune infiltrates were positively correlated with poor prognosis, indicating that infiltrating immune cells contribute to poor ccRCC results.

The collagen family (COL1A2, COL1A1, COL6A3, and COL5A1), DCN, FBLN1, and POSTN were the most abundant components of the tumor ECM. Through its effects on cancer cells and stromal cells, the ECM can increase many of the cancer hallmarks, such as angiogenesis induction (Mammoto et al., 2011), invasion, and metastasis activation (Leight et al., 2012; Pickup et al., 2015). Decorin (DCN) can play a proangiogenic role by facilitating the adhesion and migration of endothelial cells on type I collagen (Semler et al., 2010). In particular, decorin mediates adhesion by binding to integrin α2β1 and promoting the interaction between integrin and collagen (Davies et al., 2001). It is interesting that decorin has also been involved in downregulating models of the E-cadherin binding partner β-catenin in *in vitro, in vivo*, and xenograft experiments (Bi et al., 2008; Goldoni et al., 2009; Buraschi et al., 2010). Loss of E-cadherin promotes metastasis by inducing disaggregation of cancer cells, activating specific downstream signal transduction pathways, and causing epithelial–mesenchymal transition (EMT), which facilitate metastasis. In various aspects of tumor cells, such as cell motility (Lee et al., 2005), cell proliferation (Cheng et al., 2008), apoptosis, and angiogenesis (Xie et al., 2008), fibulin-1 (FBLN1) was reported as a novel ECM protein. Activation of the epidermal growth factor receptor (EGFR) is a vital oncogenic signaling regulator for the invasion and metastasis of cancer cells (Normanno et al., 2006). It has been shown that FBLN1-mediated EGFR signaling regulates cell adhesion and motility (Alexi et al., 2011; Bakker et al., 2017).

Overall, our systematic genomic and transcriptomic analyses showed that shared DEGs could play a vital role in ccRCC tumor aggressiveness. A total of 20 shared DEGs and 10 hub genes were identified in this study, and seven core genes (COL1A2, COL1A1, COL6A3, COL5A1, DCN, FBLN1, and POSTN) were associated with immune infiltrates and worse overall survival. To prove this hypothesis, we need to conduct a series of experimental studies to obtain more precise data on these correlations. In addition, the subsets of these genes could be used to code for secreted proteins and membrane receptors for both potential therapeutic and diagnostic targets.

## Data Availability Statement

The datasets presented in this study can be found in online repositories. The names of the repository/repositories and accession number(s) can be found in the article/Supplementary Material.

## Ethics Statement

Written informed consent was obtained from the individual(s) for the publication of any potentially identifiable images or data included in this article.

## Author Contributions

SG: conception and design. FS and HZ: administrative support. LY and XF: collection and assembly of data. SG and XJ: data analysis and interpretation. All authors: manuscript writing and final approval of the manuscript.

## Conflict of Interest

The authors declare that the research was conducted in the absence of any commercial or financial relationships that could be construed as a potential conflict of interest.

## Abbreviations

BP: biological process
CC: cell component
ccRCC: clear cell renal cell carcinoma
DEGs: differentially expressed genes
ECM: the extracellular matrix
EMT: epithelial–mesenchymal transition
FC: fold change
GEO: Gene Expression Omnibus
KIRC: kidney renal clear cell carcinoma
KM: Kaplan–Meier
MCODE: Molecular Complex Detection
MF: molecular function
OS: overall survival
TMB: tumor mutation burden.
